# Dengue virus 3 genotype I (GI) lineage 1 (L1) isolates elicit differential cytopathic effect with syncytium formation in human glioblastoma cells (U251)

**DOI:** 10.1186/s12985-023-02168-y

**Published:** 2023-09-03

**Authors:** Adriana de Souza Andrade, Sofia Oliveira Campos, Jamile Dias, Marco Antônio Campos, Erna Geessien Kroon

**Affiliations:** 1 Laboratório de Vírus, Departamento de Microbiologia, Universidade Federal de Minas Gerais, Belo Horizonte, Minas Gerais Brazil; 2 Imunologia de Doenças Virais, Instituto René Rachou, Fundação Oswaldo Cruz, Belo Horizonte, Minas Gerais Brasil

**Keywords:** Dengue virus, Flaviviridae, Syncytium, Human glioblastoma cells, Cytopathic effect

## Abstract

**Background:**

*Dengue virus* (DENV) is a *Flaviviridae* member classified into four antigenically distinct serotypes (DENV 1, 2, 3, and 4) and further subdivided genotypes. DENV3 is subdivided into four or five genotypes, depending on the classification adopted. Despite their high genetic proximity, as revealed by phylogenetic complete polyprotein analysis, DENV3 MG-20 and DENV3 PV_BR showed different neurovirulence in mice models. Our group identified six amino acid mutations in protein E, including the E62K and E123Q, which may affect interactions of hydrophobic clusters on domain II, thus leading to the observed differences in the studied viruses.

**Methods:**

Human glioblastoma cells (U251) derived from a malignant glioblastoma tumor by explant technique were infected by the DENV3 GIL1 isolates DENV3 MG-20 and DENV3 PV_BR and analyzed by plaque assays and titration, optical, immunofluorescence, and transmission electronic microscopy.

**Results:**

The two isolates showed different cytopathic effects (CPE) and fusogenic patterns, further confirmed by indirect immunofluorescence. Transmission electron microscopy revealed intense cytopathic effects in DENV3 MG-20 infected U251 cells, displaying endoplasmic reticulum hypertrophy and turgid vesicles with proteins and multiple viruses, distinct from DENV3 PV_BR infected cells. It is hypothesized that the different amino acids in the DENV3 MG-20 isolate are related to an increased membrane fusion ability in viral infection, thus facilitating immune system evasion and increased chances of central nervous system cell infection.

**Conclusion:**

These results emphasize the biological differences between the isolates, which could be a critical factor in host-virus interaction and severe dengue development. Our study presents comparative results of highly similar isolates with the potential to generate more subsidies for a deeper understanding of the DENV pathogenesis. The neurotropism of the isolate DENV3 MG-20 (belonging to the DENV3 GI L1 genotype) showing infection of nervous system cells (U251) could contribute to understanding neurological dengue disease.

## Background

*Dengue virus* (DENV) is an arbovirus member of the *Flaviviridae family* and *Flavivirus* genus. The virus is classified into four antigenically distinct serotypes (DENV 1, 2, 3, and 4) and subdivided into genotypes [1]. Dengue is a significant global disease caused by DENV, with an incidence estimated at 390 million cases per year, in which 60 million develop symptoms [2, 3]. Due to the dramatic increase in DENV incidence and spread, symptoms such as central nervous system (CNS) involvement have increased [4]. The spectrum of illness ranges from asymptomatic to dengue or severe dengue [5]. Dengue clinical findings include nausea, vomiting, rash, muscle, and joint pain, as alarm signals leukopenia, abdominal pain, persistent vomiting, clinical fluid accumulation, mucosal bleeding, lethargy, and postural hypotension [6]. Complications can lead the individual to death, including severe plasma leakage, respiratory distress accumulation, severe bleeding, shock, impaired consciousness, and severe organ impairment. Severe organ impairment includes hepatitis, myocarditis, pancreatitis, and encephalitis [5, 6]. The evolution to severe conditions is not well elucidated. However, it is understood that host factors such as abnormal immune response, homeostatic disorder, and intrinsic characteristics of the infecting virus strains are essential [7].

DENV genotypes have been associated with different clinical manifestations, including severe forms of the disease [8, 9]. Thus, depending on viral isolate characteristics such as tropism, ability to evade the host’s immune response, and biological barriers evasion, it is possible to observe differences in vivo and in vitro infection and viral pathogenesis [10–12].

Between 2002 and 2006, a DENV3 genotype III outbreak in Brazil presented many cases [13]. Simultaneously, reports of DENV3 G1L1, also known as DENV3 G genotype V, according to Wittke et al., 2002, occurred in this outbreak [14]. However, the DENV3 genotype I (GI) lineage 1 (L1) (DENV3 G1L1) has only been reported in some states, such as Minas Gerais, Rondônia, and Pará [8, 15]. In a previous study, we compared the DENV3 GIL1 isolates from Minas Gerais-MG (DENV3 MG-20) and Rondônia-RO (DENV3 PV_BR). The in vivo analysis and in vitro assays of heparan sulfate adhesion showed differences between these isolates, although they belong to the DENV G1L1 [12]. DENV3 MG-20 was isolated from a fatal meningoencephalitis case from MG [8], with reproduction of neurovirulence in a murine model [12, 16], while DENV3 PV_BR [12] and other isolates from RO showed no neurovirulence in mice [17].

Here, we report using plaque unit forming titration, qPCR, immunofluorescence, and transmission electron microscopy that these isolates infect cells derived from a malignant human glioblastoma (U251 line). However, DENV3 MG-20 infected these cells with syncytium formation and cell death, while DENV3 PV_BR infected cells without syncytium formation or any other cytopathic effect (CPE) evidenced by optical microscopy. These results emphasize the difference in biological characteristics of these isolates, which could be a critical component in the interaction with the host cell and the development of severe dengue.

## Methods

### Virus and cells

DENV3 MG-20 (GenBank Accession number EF625835.1) is an isolate from the Laboratório de Vírus collection, Universidade Federal de Minas Gerais. DENV3 PV_BR (ACD85885.1) was isolated from a patient’s serum sample from Porto Velho, Rondônia State, Brazil. Professor Luiz Tadeu Figueiredo kindly provided it from USP/Ribeirão Preto. The cell lines used were C6/36 cells (ATCC CRL-1660), BHK-21 cells (ATCC CCL-10), Vero cells (ATCC CCL-81), and U251 MG Cells. The maintenance of these cell lines has been previously described [12].

### In silico assays

Phylogenetics was analyzed using 93 DENV3 complete protein sequences retrieved from GenBank (https://www.ncbi.nlm.nih.gov/genbank/). Sequences were aligned using the Clustal Omega server available from the European Bioinformatics Institute (https://www.ebi.ac.uk/Tools/msa/clustalo/). Phylogenetic trees were reconstructed using the neighbor-joining method, with 1000 bootstrap replicates. Tree construction was carried out through the MEGA 7 software. Partial sequences with high similarity to DENV3 GIL1 (up to 94%) were surveyed using GenBank BLASTP/N (server on www.ncbi.nlm.nih.gov/blast). Molecular and spatial analysis of DENV3 GIL1 was done using the software PyMol 4.6.0 and Chimera 1.14. The 4GSX model from the protein data bank (PDB) was used for the analyses. The modeling was done as described in reference 12. Hydrophobicity analysis was done according to Eisenberg’s scale, and the protein molecules’ color was generated using the Color h script.

### Plaque assays and titration

For phenotypic characterization in C6/36 cells, 9 × 10^5^ cells were seeded in 6-well plates, incubated for 24 h, and then infected DENV3 GIL1 MG-20 or DENV3 PV_BR samples. After 1 h adsorption, 1% carboxymethylcellulose (CMC) (Synth, Brazil) was added to the wells, dissolved in Leibovitz medium (Gibco, USA) supplemented with 5% fetal bovine serum (FBS; Cultilab, Brazil), 20 μg/ml streptomycin (Sigma–Aldrich, USA), 100 IU/ml penicillin (Cultilab, Brazil), and 2 μg/ml amphotericin (Sigma–Aldrich, USA), and the cells were incubated for 7 days at 37 °C. The cell monolayers were then fixed in 10% formalin and stained with a 1% crystal violet solution. Titration assays were done with 2.5 × 10^5^ BHK-21 cells in each 6-well plate. After viral adsorption, cells were incubated with 0.5% CMC in Medium (Gibco, Brazil) at 37 °C and with 5% CO_2_. After 7 days, cell monolayers were fixed and stained as described above, and viral plaques were counted to determine the titers (PFU./ml).

### Replication curve

U251 cells were infected with DENV3 MG-20 and DENV3 PV_BR samples at 0.1 MOI (multiplicity of infection). Supernatant and cell lysate (lysis heat shock) were collected from each well at 24 h, 48 h, 72 h, and 96 h post-infection. The viral replication curve was made by titration (described above), and the RNA replication curve was generated by real- time qPCR data. Titration was performed in triplicate. As a negative control, uninfected cells were used. RNA was extracted using the RNeasy Mini Kit (Qiagen, USA) extraction with 140 μl of the culture supernatant or harvested cell culture (previously rinsed with medium). Alm et al., 2015 [18] described the reverse-transcriptase PCR reaction. The target was the coding region of the NS1 protein, and as a constitutive gene, primers for b-actin were used. The comparative Ct method was used for data analysis, applying the mathematical form of ΔCt (Applied Biosystems guide). Results were plotted as units of arbitrary genome copies. All reactions were performed in triplicates and analyzed in GraphPad Prism 6.

### Optical microscopy and immunofluorescence

Under sterile conditions, 24-well plates with coverslips were seeded with U251 (70,000) and C6/36 (150,000) cells. After 24 h, monolayers were infected with 0.1 MOI of the DENV3 MG-20 and DENV3 PV_BR viruses. The adsorption was followed by 4 days of incubation. Then, cells were washed with PBS and fixed with cold acetone. The assay used 4G2 as a primary antibody (1:100) and Alexa 488 conjugated anti-mouse IgG (Sigma, EUA) 1:200 as a secondary antibody. An additional 30 min incubation was performed to stain the nuclei with DAPI. Coverslips were counterstained with Evans Blue. After mounting, the coverslips were observed under the DI-FL03 and Axio Imager Z2-ApoTome 2 Zeiss microscope models. Green fluorescent staining for virus identification was observed under blue light (wavelength 519 nm), and the cell nucleus was identified by sky blue staining (red light, wavelength 358 nm). Uninfected cells were kept as a control.

### Transmission electronic microscopy

U251 cells were grown on coverslips to 90% confluence and infected with DENV3 PV_BR or DENV3 MG-20 at 1 MOI in DMEM with antibiotics and 2% FBS. Uninfected U251 cells were kept as a control. After 4 days of incubation, cells were harvested and fixed with 1 ml of 2.5% glutaraldehyde (Sigma) in 0.1 M phosphate solution (0.052 g of NaH2PO4.H2O + 0.435 g of Na2HPO4.7H2O in 20 ml of distilled water) under stirring for two hours. The cell layer was recovered, transferred to a 1.5 ml microtube, and centrifuged at 3000 g for 5 min. The glutaraldehyde phase was withdrawn from the experiment, and 200 μl aliquots of phosphate solution were added to the tubes. The samples were stored at 4 °C until they were sent to the UFMG Microscopy Center. Slides were read using the transmission electron microscopy model Tecnai G2-20 - SuperTwin FEI − 200 kV.

## Results

### Despite different neurovirulence patterns, there is a high similarity of DENV3 GIL1 isolates in phylogenetic analysis

**Fig. 1:**
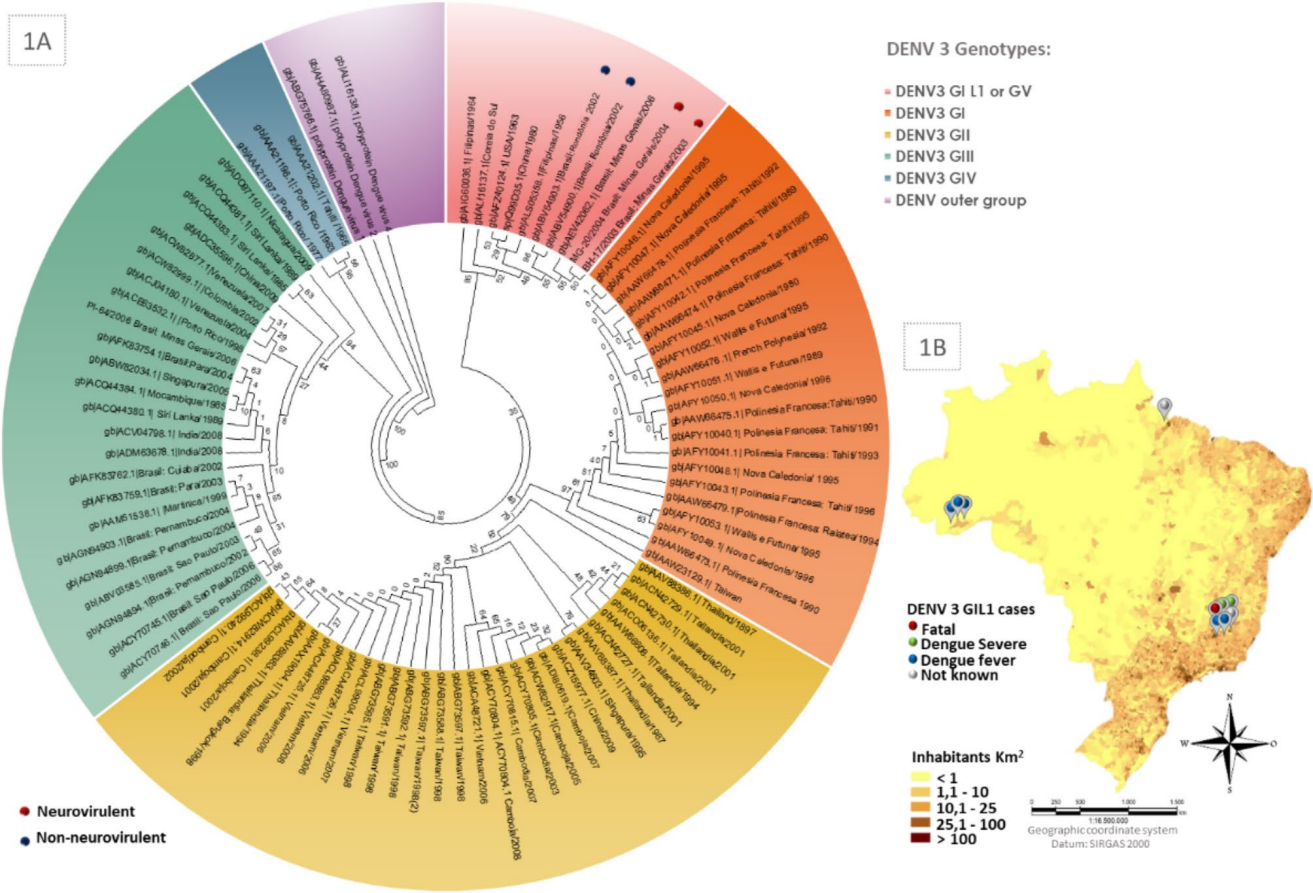
Phylogenetic and geographic distribution of DENV3 G1L1 sequences. **1A** Neighbor-joining analysis was performed using 3395 amino acids corresponding to DENV3 polyprotein from 93 samples of DENV3. Bootstrap values ​​were indicated in percentage for the branches. The DENV3 GIL1 clade (red) was classified following Lancioti et al. (1994) [20] or DENV3 GV (Wittke et al., 2002) [14]. The red markings correspond to the sequences identified in southeastern Brazil (Minas Gerais) related to neurovirulence, while the blue markings correspond to sequences identified in the Amazon region (Rondônia). The phylogenetic analysis was performed using the MEGA7 software. **1B** Brazilian territory map showing partial or complete sequences of DENV3 GIL1 distribution related to the different demographic densities. The locations of 10 complete or partial DENV3 GIL1 sequences found in Brazil and identified in the NCBI database are flagged. The DENV3 MG-20 (red dot) represents a fatal case

Phylogenetic complete polyprotein analysis shows the genetic proximity of the studied viruses. As shown in Fig. 1A, DENV3 has clades corresponding to 4 or 5 genotypes, depending on the classification adopted. This study identified ten DENV3 G1L1 complete polyprotein sequences of this rare lineage in Genbank. Four have a known neurovirulent profile in the mouse model. Two were previously described as non-neurovirulent; gb|ABV54903|, gb|ABV54900 [17], and two others as neurovirulent DENV3 MG-20 gb|EF625832.1.1|, BH-17 gb|ABR13854.1 [12, 19]. These viruses are very close phylogenetically and are grouped in a specific clade containing the DENV3 GIL1 (or DENV3 GV) prototype sequences H87 (ALS05358.1). This phylogenetic tree was built using a neighbor-joining algorithm and analyzing amino acids of the 93 complete polyproteins DENV3 sequence. Divergences between the complete polyprotein sequence of MG isolates DENV3 MG-20, BH-17 gb|AEV42062|, and RO isolates gb|ABV54903|, and gb|ABV54900| have a maximum value of 0,008. After identifying the DENV3 GIL1 sequences, this information was used as a template for identifying partial sequences from other viruses of the same genotype using BLAST. We found 10 sequences related to DENV-3 GIL1 from Brazil in NCBI. Among them, 6 were from Minas Gerais, 3 from Rondônia, and 1 from Pará (Fig. 1B). Among the isolates from Rondônia, we identified the PV_BR E sequence (gb|ACD85885.1|) in the NCBI data bank, and its characterization showed attenuated neurovirulence in mice [12].

**Fig. 2:**
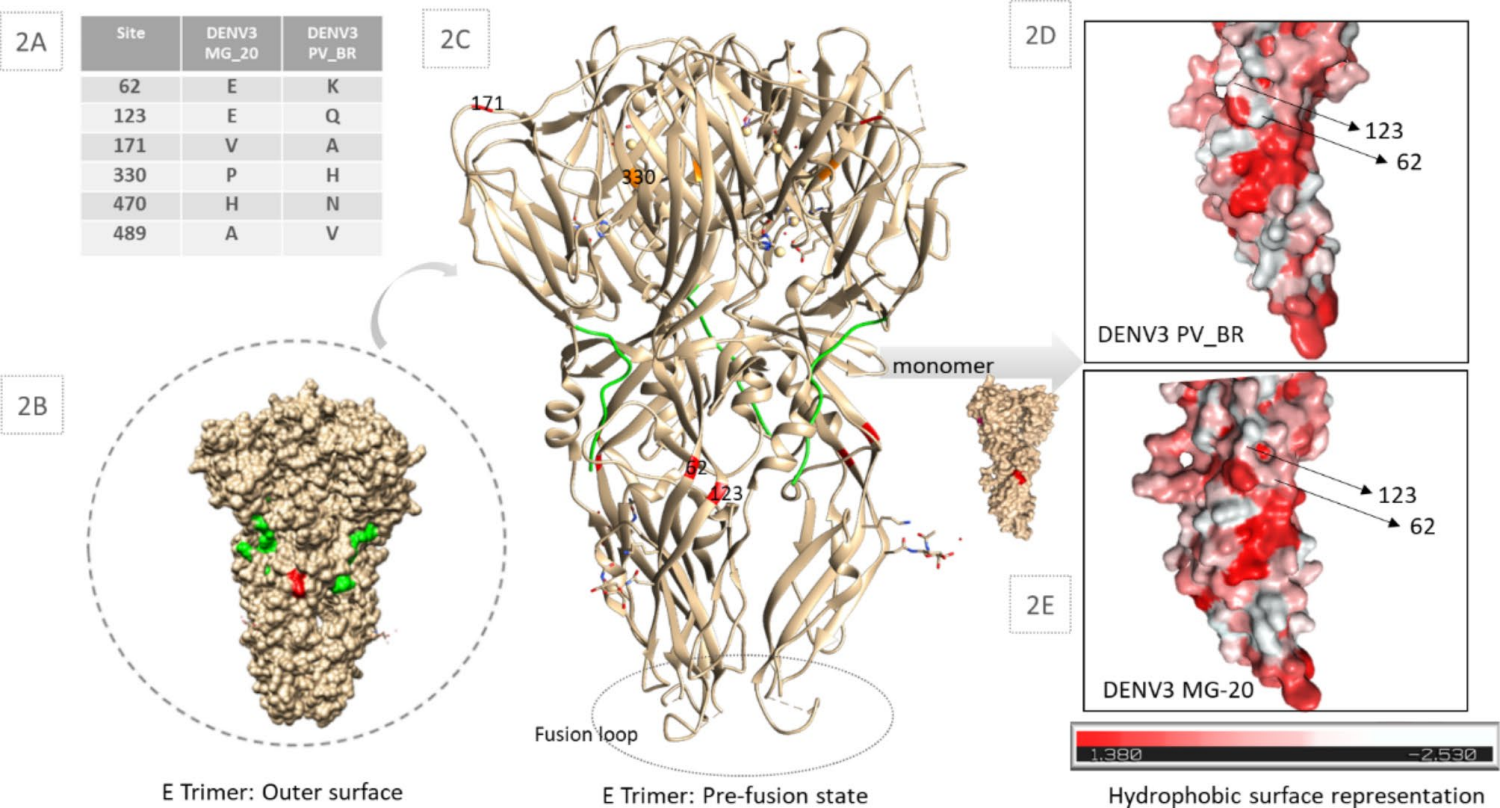
Amino Acid Divergences and Structural Analysis of DENV3 MG-20 and DENV3 PV_BR. **2A**. Mutations Table. **2B**, and **C**. DENV3 G1 structure modeled using the 4GSX template from Protein Data Bank. The structure is presented in a surface representation, enabling observation of mutations on the outer face of the protein (**2B**). In **3C**, the structure is presented in a cartoon representation, allowing observation of mutations within the internal protein structure. The structures were visualized using Chimera 1.14. software. Orange dots represent conservative changes, and red dots represent non-conservative changes at positions 62 and 123. The green region corresponds to the stem. **2D**. Three-dimensional E protein monomer surface hydrophobicity representation based on Eisenberg’s scale. The representation illustrates changes in hydrophobicity for amino acid 62. The visualization was performed using PyMol 4.6.0 software and the Color h script

Despite the high E similarity (99,98%) between DENV3 MG-20 and DENV3 PV_BR, 6 different amino acids (Fig. 2A) may potentially play an important role in the observed differences between the viruses. These mutations are distributed in the E DENV fusogenic protein. The mutation V171A is located in Domain I, P330N internally in Domain III, and H470N and A489V are outside the 4GSX model in the transmembrane region (Fig. 2B, and C). The mutations E62K and E123Q are localized in Domain II, and spatially adjacent on the outer face of the protein. As shown in the spatial models, these mutations are non-conservative, resulting in charge changes and hydrophobic variation. These mutations are located near a hydrophobic cluster of the protein, as indicated in red, based on Eisenberg’s scale (Fig. 2D, and E).

### DENV3 GIL1 isolates infect human glioblastoma cells (U251) with different patterns of cytopathic effect (CPE)

**Fig. 3:**
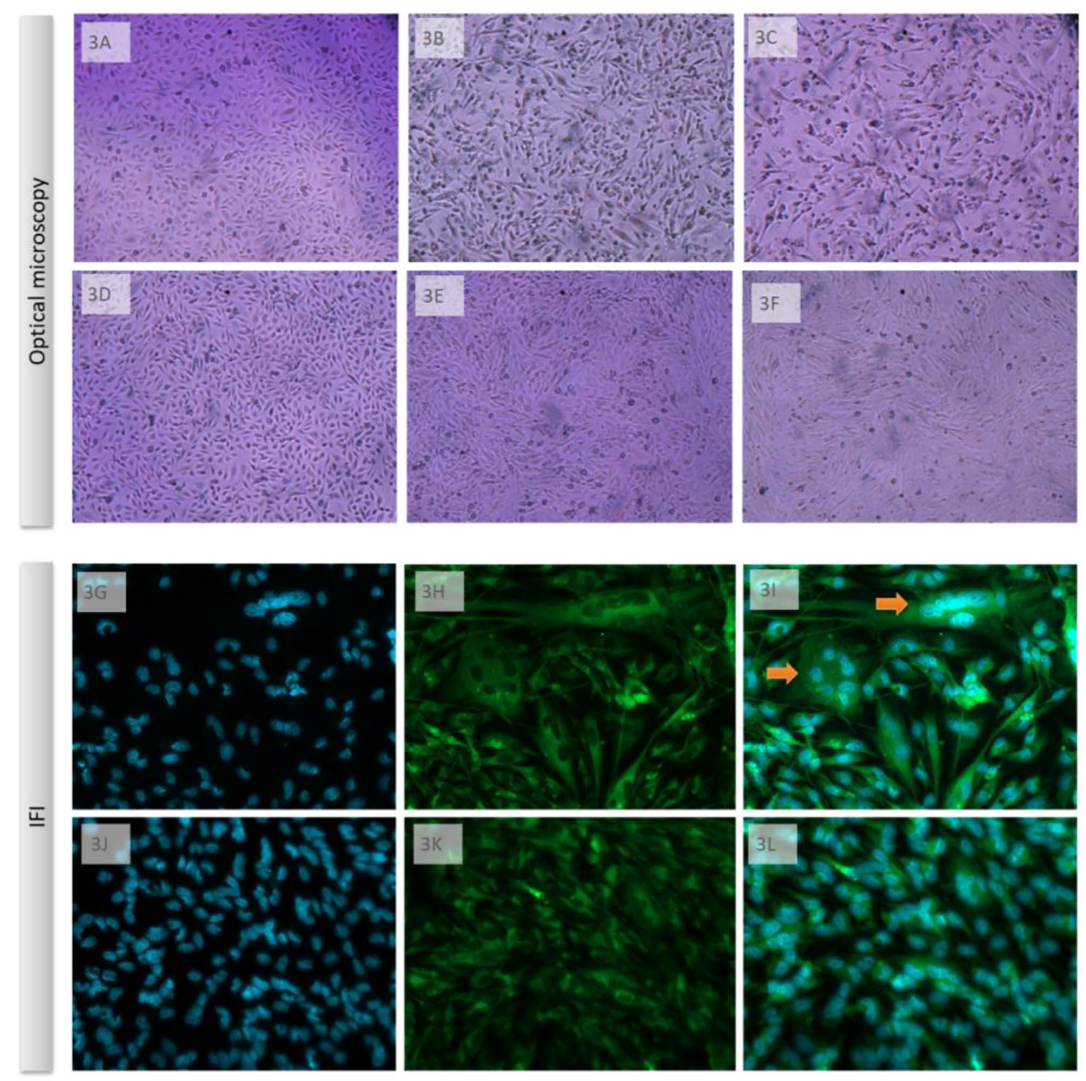
Different patterns of CPE observed in glioblastoma cells (U251) infected with DENV3 GIL1 isolates. U251 cells were infected with MOI 0.1 of DENV3 MG-20 or DENV3 PV_BV, and photos were taken at 0, 3, and 6 DPI, respectively, as shown in panels **3 A-C** for cells infected with DENV3 MG-20 and **3D-F** for cells infected with DENV3 PV_BV. Photos from optical microscopy were taken under 100x magnification. **3G-L**: For immunofluorescence, cells were infected and at 4 DPI treated Alexa fluor 488 as secondary antibody for identification of dengue envelope/4G2 monoclonal antibody complex (green color), and with DAPI to identify the nucleus (blue color). **3G-I.** Cells infected with DENV3 MG-20. **3J-L**. Cells infected with DENV3 PV_BR. The immunofluorescence photos were taken under 200x magnification. Arrow indicates syncytium formation

In U251 cell culture, a human glioblastoma lineage, DENV3 MG-20 infection exhibited a cytopathic effect, whereas DENV3 PV_BR did not such an effect, as observed by optical microscopy. DENV3 MG-20, the neurovirulent virus, displayed a CPE with visible cell lysis beginning at three days post-infection (DPI) and resulting in the destruction of the cell monolayer by six days (MOI 0.1) (Fig. 3A-C). The DENV3 PV_BR, under the same culture condition and using the same MOI, did not exhibit any CPE (Fig. 3D-F), even after 15 days. Using indirect immunofluorescence (IFI), we could observe that the DENV3 MG-20 also showed syncytium formation on U251 cells in addition to the lysis effect. However, CPE was not observed in cells infected by DENV3 PV_BR under IFI observation (Fig. 3J-L).”The indirect labeling with a fluorescent antibody allowed us to confirm cell infection, even though there was no visible CPE with DENV3 PV_BR. Plaque assays were used to determine the viral titer on BHK-21 cells, and comparative Δct PCR was employed to analyze genomic RNA.

**Fig. 4:**
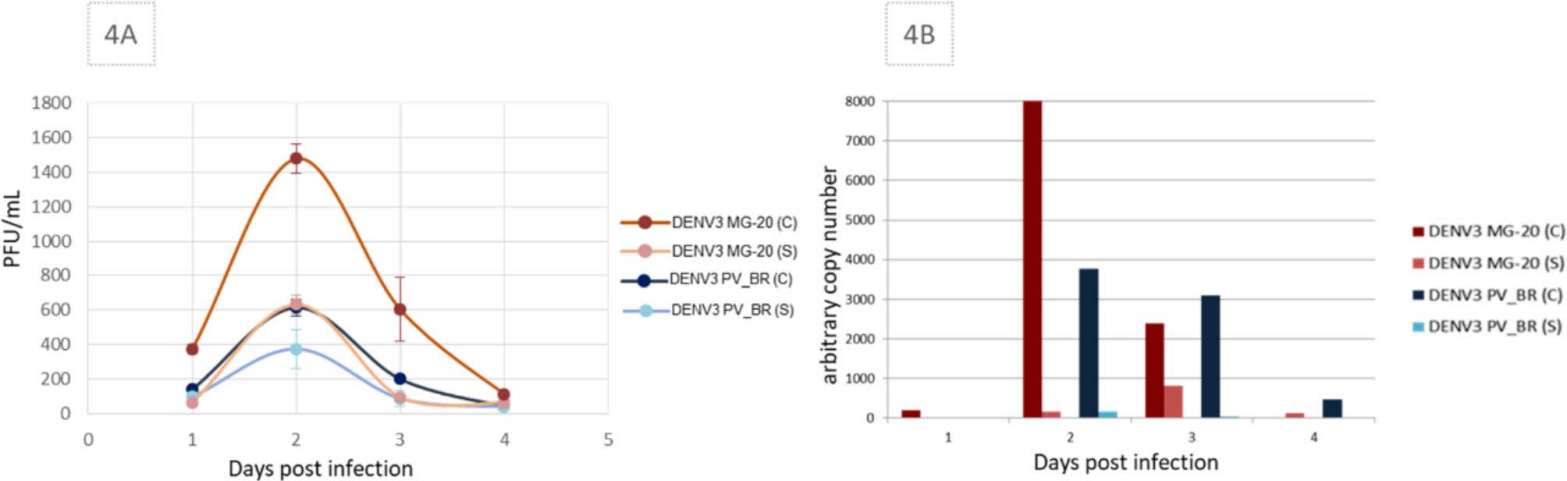
DENV3 MG-20 and DENV3 PV_BR replication in U251 cells. **4A** Viral titers by plaque-forming units (PFU) per ml from the supernatant (S) and cell lysate (C) at 1 to 4 DPI. **4B** the arbitrary copy units by RT-PCR.

The analysis of virus titer in the supernatant and cell lysate at 1 to 4 DPI for both viruses revealed that the virus titers were higher in the cell lysate than in the cell supernatant (Fig. 4A). Examining the replication curve, we observed that from 3 DPI onwards, there was a sharp reduction in DENV3 MG-20 titers. The highest titers in the cell lysates were observed at 2 DPI for both viruses, with 1,4 × 10^3^ and 6,2 × 10^2^ PFU/ml titers for DENV3 MG-20 and DENV3 PV_BR, respectively. The analysis of the arbitrary Δct PCR showed a similar result to virus titration (Fig. 4B), indicating a milder infection but with a prolonged presence of DENV3 PV_BR. In DENV3 MG-20 infection, viral RNA was detected on the 2 DPI in, followed by a sharp drop on consecutive days. A possible mechanism of cell injury is evident starting from the 3 DPI.

### C6/36 mosquito cell lines also show different cytopathic patterns after infection with DENV3 G1L1 isolates

**Fig. 5:**
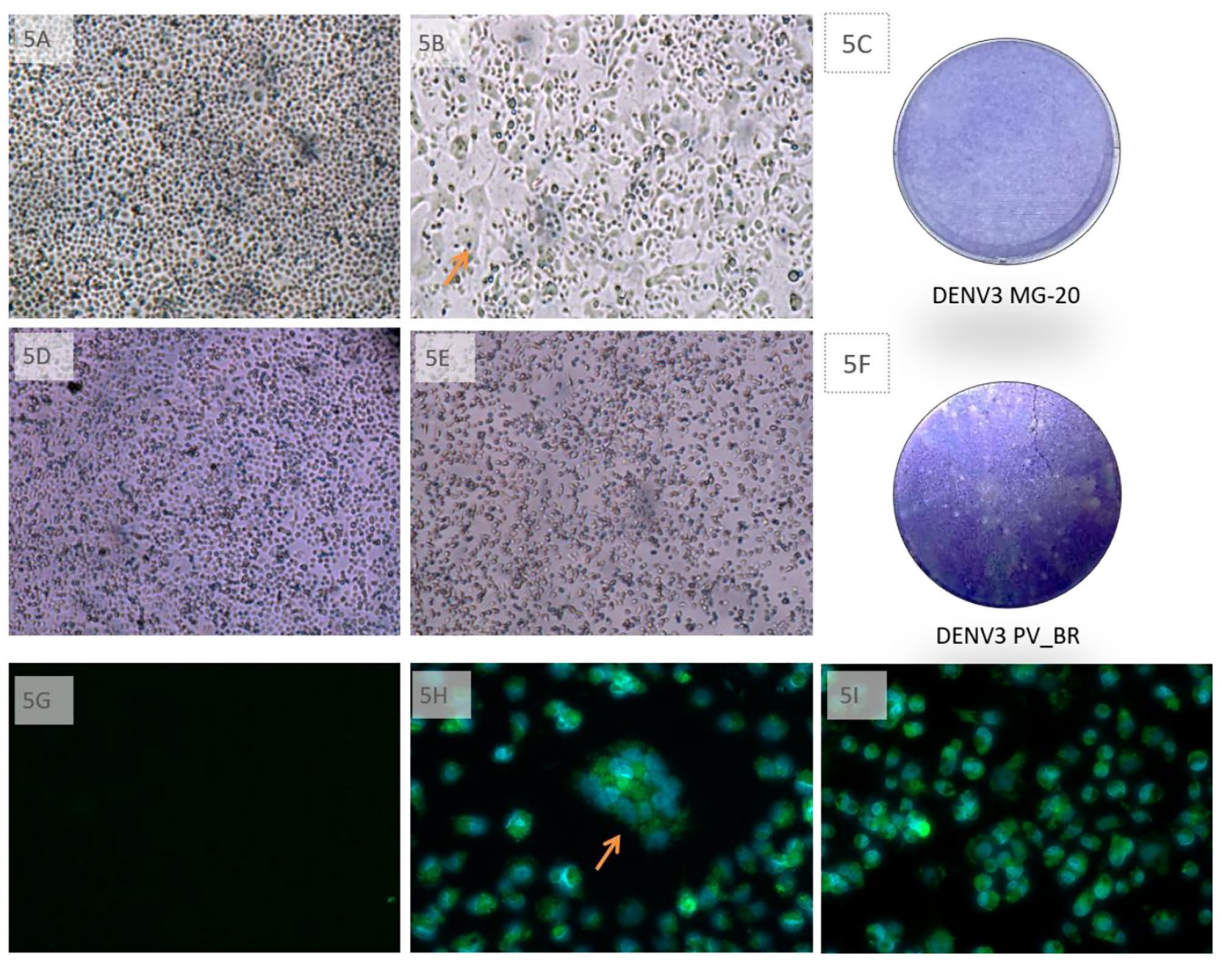
Different CPE patterns of DENV3 GIL1 infection in mosquito cells. C6/36 cells were infected with MOI 0.1 of DENV3 MG-20 or DENV3 PV_BV, and photos were taken at 0 and 6 DPI, respectively, as shown in panels **5A-B** for cells infected with DENV3 MG-20 (arrow indicates syncytia formation) and **5D-E** for cells infected with DENV3 MG-20. **5C** and **5F.** Infected C6/36 cells with DENV3 MG-20 and DENV3 PV_BR, respectively, diluted to 10^− 4^ and overlayed with CMC were stained at 6 DPI with crystal violet. **5G-I**. For immunofluorescence, cells were infected and at 6 DPI treated Alexa fluor 488 as secondary antibody for identification of dengue envelope/4G2 monoclonal antibody complex (green color), and with DAPI to identify the nucleus (blue color). **5G** Negative staining control. **5H** DENV3 MG-20 cell infection with syncytium formation indicated by an arrow. **5I** DENV3 PV_BR cell infection

In C6/36 cells, DENV3 MG-20 exhibits a classic CPE described for DENV, with syncytia formation (Fig. 5A, and B). On the other hand, DENV3 PV_BR shows a lytic effect on these cells (Fig. 5D, and E), which can be observed even by plaque phenotype analysis (Fig. 5F). IF microscopy of cells infected with DENV3 MG-20 confirms syncytia formation, which was not detected in cells infected with DENV3 PV_BR (Fig. 5G-I). In Vero cells and the murine neuronal cell (Neuro 2 A) lineage, no CPE was observed by optical microscopy (data not shown).

### Both viruses infect cells, but DENV3 MG-20 is the neurovirulent one

**Fig. 6:**
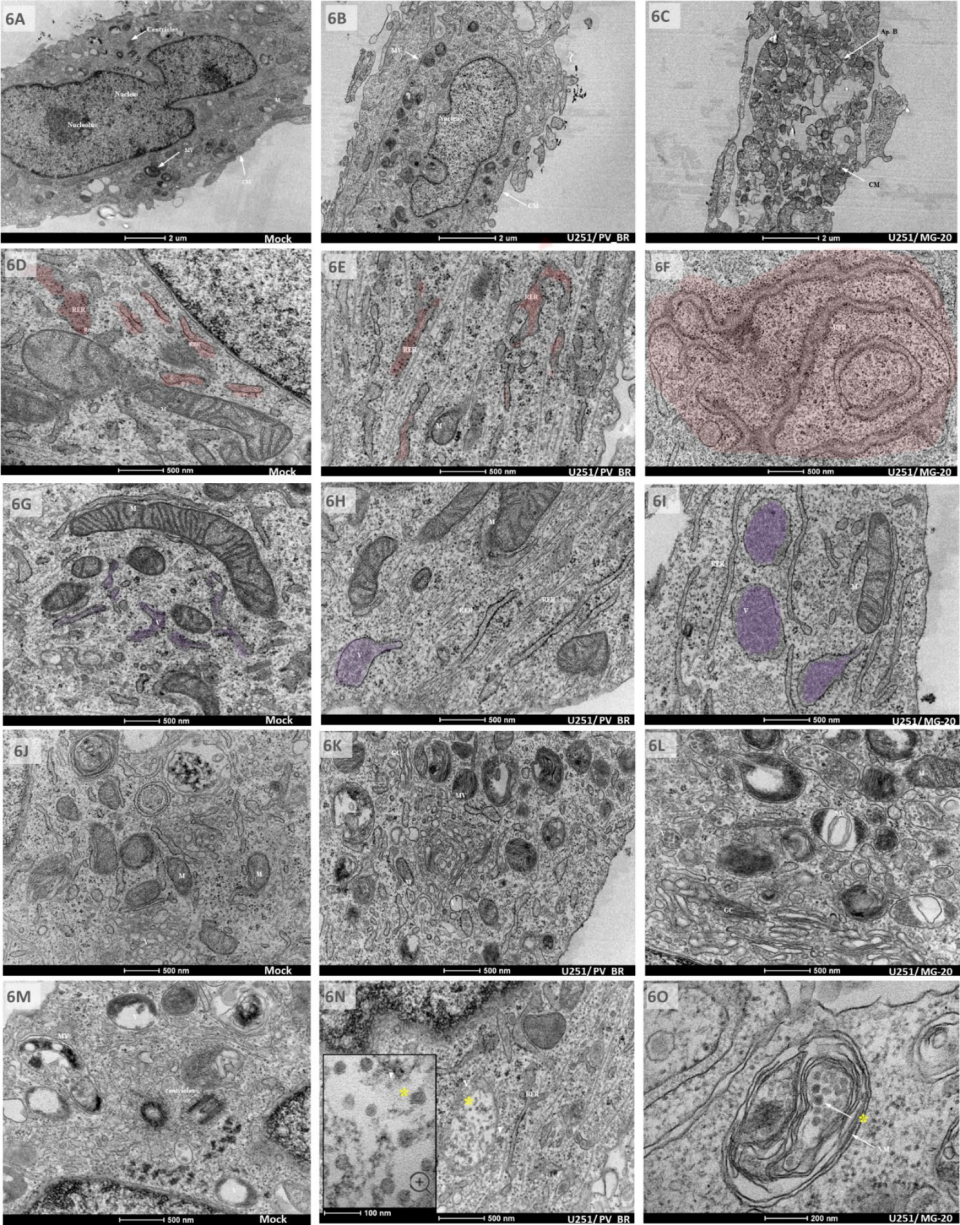
Transmission electron microscopy (TEM) analysis. Human glioblastoma cells (U251) infected with DENV3 GIL1 isolates was observed at 1 MOI on the 4th day. Cell morphology and visualization of membranes (2 μM scale bar) (**6A–C**) showed a constriction in the nucleus, the presence of a pair of centrioles, an intact cell membrane (CM), and a healthy-looking cell structure in the mock-infected cell (**6A**). DENV3 PV_BR (**6B**) was observed to have integral membranes, while DENV3 MG-20 (**6C**) was found to have multiple membranous compartmentalization indicating a possible process of cell death by apoptosis (Ap. B – Apoptotic bodies) and damaged CM. At 500 nm scale bar, the transmission electron microscopy (TEM) analysis of the mock-infected cell (**6D**), DENV3 PV_BR (**6E**), and DENV3 MG-20 (**6F**) revealed a regular morphology of ribosomes (RER) with small electrodense particles adhered to the reticles; mild rough RE hyperplasia in DENV3 PV_BR; and a region of intense RER hyperplasia in DENV3 MG-20. At 500 nm scale bars, the visualization of vesicles with proteins showed regular size of vesicles in the mock-infected cell (**6G**), turgid vesicles with organic molecules in DENV3 PV_BR (**6H**), and a large flow of dilated vesicles with large amounts of organic molecules in DENV3 MG-20 (**6I**). These suggested potential virus morphogenesis regions. At 500 nm scale bars, the intense activity due to the high presence of organelles and reticulum in the mock-infected cell (**6J**); intense metabolic activity with excess organelles and membranes indicating a putative region of viral synthesis in DENV3 PV_BR (**6K**); and high metabolic activity with excess of organelles, membranes, vesicles and Golgi complex in DENV3 MG-20 (**6L**), suggesting that this area is a more intense region of viral synthesis than that of DENV3 PV_BR. At 500 nm scale bars, observation of vesicle structures revealed misshapen and disorganized filling in the mock-infected cell (**6M**), vesicles with electron-dense particles, circular, with an icosahedral core suggestive of DENV3 viral particles in DENV3 PV_BR (**6N**), and multi-membranous vesicles containing electron-dense particles Dengue virus-like in DENV3 MG-20 (**6O**). The geometries and sizes were compatible with those described for the DENV (at 200 nm scale bar), yellow asterisk indicates virus

Observing viral replication and differences in CPE in human nervous system cells raised questions about the intracellular responses related to neurovirulence. To investigate further, we analyzed the U251 cells using transmission electron microscopy. The cells were infected at 1 MOI and fixed 4 DPI. In uninfected cells, we observed a normal morphology with a visible cytoskeleton and nucleus presenting regions of well-condensed chromatin. Several organelles, such as mitochondria, endoplasmic reticules, and some multi-membrane vesicles, displayed a normal appearance and distribution. Centrioles were visualized, indicating that cells maintained their replication cycle (Fig. 6A). In DENV3 MG-20 (Fig. 6C), we observed several vacuolized cells, indicating a stage of degeneration suggestive of apoptosis. The endoplasmic reticulum appeared hypertrophic and showed an increase in the flow of and size of vesicles filled with a large amount of protein (Fig. 6F, and I). Notably, in Fig. 6L, there was an intense and large number of transit vesicles containing viral particles near several membranous organelles, endosomes, and Golgi complexes, suggesting a region of viral morphogenesis. Furthermore, in some of these vesicles, it was possible to observe electrodense circular particles with size and characteristics suggestive of DENV (Fig. 6O).

In cells infected with DENV3 PV_BR, we also could observe hypertrophic endoplasmic reticula and an increased presence of vesicles with proteins compared to the control cells. Some multi-membranous vesicles are observed in Fig. 6B and E, and 6 H at a significant frequency compared to the control, but at a lower frequency than the cells infected with DENV3 MG-20. Viral replication apparatus and particles were also identified (Fig. 6K), suggesting the virus replication, even though no CPE was visible under the optical and IF microscopy in U251 cells. Notably, no cells in advanced degenerative processes were found in DENV3 PV_BR infection.

### Syncytium formation and neurovirulence: could they be interconnected factors to the developing neurotropic dengue disease?

**Fig. 7:**
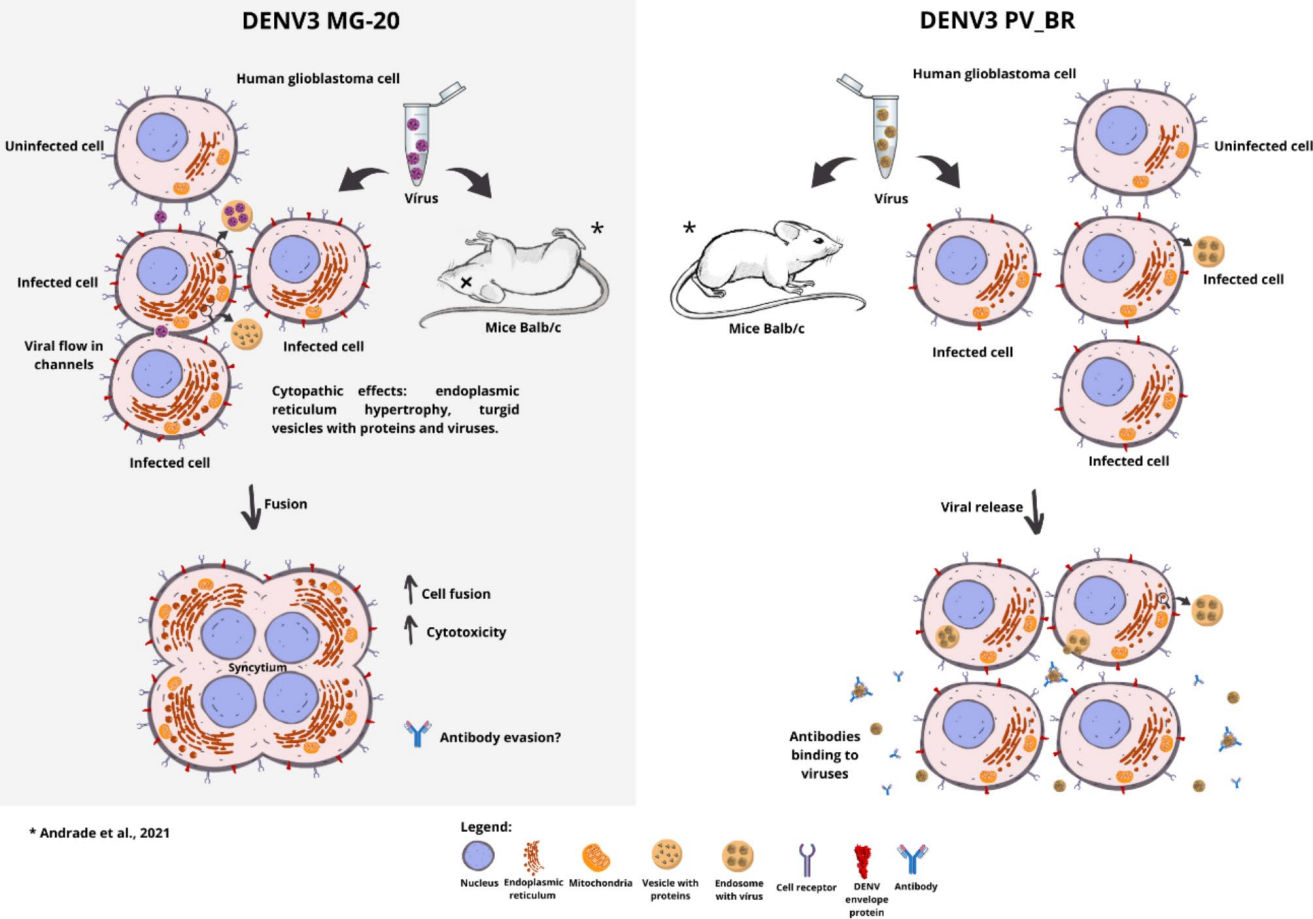
DENV 3 MG-20 and DENV 3 PV_BR infection models. DENV3 MG-20 was isolated from a fatal human case with CNS involvement, and this pathogenic behavior was reproduced in a mouse model, leading to animal death with CNS disease. When inoculated into human CNS cells (glioblastoma), it caused an intense cytopathic effect with endoplasmic hypertrophy, an intense flux of turgid vesicles with viruses and viral components leading to cell-to-cell fusion, syncytium, and cell death. We hypothesize that the neurovirulence of this virus is related to reduced titer in the extracellular environment and escape from the humoral response. In contrast, DENV3 PV_BR presented discrete CPE, as observed in TEM, on CNS cells but not leading to syncytium formation. Mice infected with DENV3 PV_BR did not have any signs of neurovirulence. We hypothesized that the balance between humoral and cytotoxic responses in this infection could impact the dissemination of the virus

We proposed a model to explain DENV neurovirulence based on our results. We used two viruses isolated from naturally infected dengue cases. DENV3 PV-BR was isolated from a patient with dengue as described, and DENV3 MG-20 was isolated from a fatal case with CNS involvement. This pattern was reproduced in mice experiments, as DENV3 MG-20 caused death by CNS disease (meningoencephalitis). On the other hand, DENV3 PV_BR does not show lethality in mice [12]. When we infected CNS human cells with DENV3 MG-20, it caused a robust CPE with syncytium formation, and no CPE was observed in cells infected with DENV3 PV_BR. However, the mechanisms related to neurovirulence are unknown, and some hypotheses have already been discussed as evasion of the humoral response and increased cytotoxicity. Our results contribute to this discussion by connecting the syncytium formation observed in human CNS cells infected with DENV3 MG-20 to a hypothesis of immune evasion (Fig. 7). This suggests that the formation of syncytia may be a strategy employed by the virus to escape neutralizing antibodies, contributing to its enhanced neurovirulence.

## Discussion

The DENV3 genotypes classification is controversial and includes 4 or 5 genotypes [14, 21]. The DENV3 GIL1 has a rare circulation and was first identified in Brazil in 2003 [8]. Despite infrequent circulation, many severe cases associated with this lineage were observed, including neurological symptoms [8, 15, 16, 20]. Using the phylogenetic construction of the DENV3 polyprotein, we found many complete DENV3 GIL1 sequences from GenBank without prior genotype identification (Fig. 1A). These sequences are grouped with the H-87 DENV3 GIL1 (GV) prototype, showing the similarity of these sequences with the Brazilian ones. Using the BLAST/P tool, we found partial sequences of this genotype. We identified information regarding geographic distribution, clinical symptoms, disease severity (Fig. 1B), mutations, and molecular markers (Fig. 2A).

Many reports have associated different genotypes with different outbreaks and clinical manifestations, including an increase in severe forms of the disease [22, 23]. The DENV3 MG-20 sample was isolated from a serum sample derived from a fatal patient with neurological manifestations in 2004, Minas Gerais State (MG), Brazil [8]. The development of neurological disease has been reproduced in mice inoculated with the DENV3 MG-20 and for other isolates such as DENV3 MG-21 and DENV3 MG-25 [12, 24]. We used the DENV3 MG-20 (a human and mouse neurovirulent isolate) and DENV3 PV_BR (a non-neurovirulent isolate) as models to observe biological differences in vitro [12]. These two isolates belong to the same genotype, have high sequence similarity to each other and to several DENV3 GIL1 (Fig. 1A), and were used to reproduce the observed neurovirulent and non-neurovirulent patterns, respectively, in glioblastoma cells.

DENV, as an enveloped virus, expresses a fusogenic protein that drives the fusion of the viral envelope with cellular membranes. In the DENV, this protein is the E protein and is expressed in the virion membrane surface. E protein interacts with cell-host receptors and cell membranes and is related to cell-to-cell fusion [25]. Point differences between the two isolates were observed in the E amino acid sequence and are represented in Fig. 2A. Viral fusion proteins have been classified into three classes (I, II, III) according to their structure and fusion mechanism with the cell membranes. E DENV protein is classified as class II, characterized by three-domain architecture (DI, DII, DIII), β-sheet-based elongated ectodomain, with a ‘fusion loop’ responsible for cellular membrane interaction and virus membrane fusion (1B, 1 C). The E trimer fusional conformation shown in Fig. 2C results from the pH change inside the cell, exposing hydrophobic domains and allowing fusion between the virus-cell membranes [26]. This protein is key in the host-pathogen interaction and infection entry stage. Mutations in sites near E hydrophobic clusters could impact the exposure of these domains and change the virus’s fusional properties [27]. We do not know the real impact of the amino acid 62 and 123 mutations on the fusional properties of E DENV3 GIL1 samples (Fig. 1F and G). However, it drew our attention that these non-conservative mutations are close to hydrophobic clusters, including a fusion loop.

The infected human glial cell line (U251) shows different CPE for these two isolates. By optical microscopy and IFI, we identified that DENV 3 MG-20 induces syncytium formation besides being lytic,. The syncytial U251 cells presented ring nuclei surrounding the central cytoplasm. Syncytium formation is identified for other viruses, such as varicella-zoster virus in glial cells [28]. DENV 3 PV_BR also showed tropism to these cells, initially observed through IFI. No morphological changes were observed by light microscopy and IFI in this case (Fig. 3). Viral titration shows the presence of infective progeny in both isolates, with peaks at 2 DPI. However, a rapid drop in viral replication occurs, especially for the cells infected with the DENV3 MG-20. The DENV 3 PV_BR concentration in the culture supernatant was constantly low and proved undetectable after 4 DPI (Fig. 4). Differences in infection productivity have been related to negative-strand viral RNA and protein accumulation and infectious particle titers [10].

As shown in Fig. 5, different CPEs can also be observed in C6/36 mosquito cells. DENV3 MG-20 infection showed a fusogenic effect, while the DENV 3 PV_BR infection markedly affected cell morphology. Although both viruses compromise the integrity of the monolayer, it is visible in the C6/36 plaque assay with the CMC (Fig. 5F), the lytic capacity of DENV3 PV_BR compared to the other isolate. The classic model to explain flavivirus cell-to-cell fusion is based on C6/36 infection. Fusion activity is mediated by virions attached simultaneously with two cells (fusion-from-without, FFWO) or viral protein incorporated or expressed on the surface of infected cells (fusion-from-within, FFWI) [29, 30].

No CPE was observed in both viruses by optical microscopy in Vero and mouse neural cells (lineage neuro 2 A) (data not shown). Although U251 and neuro 2 A are nerve cells, U251 is a stem-like cell from glial lineage, and Neuro 2 A is a stem-like cell from neuronal lineage. U251 (or U251 MG, also known as U-373 MG) used in this study, is a cell line derived from a human malignant glioblastoma multiform (GBM). Obtained by tumor explant, the U251 cell line is one of these glioma subclones and retains original features such as copy number aberrations typical of the GBM [31]. Neuro 2 A originates in a spontaneous tumor from an albino strain A mouse and can differentiate into neurons [32]. Therefore, we believe that the susceptibility of glial stem-like cells is due to their lineage functional defense characteristics, which are analogous to the function of phagocytes from other tissues (dendritic cells, macrophages), which are known to be susceptible to DENV infection.

Intracellular viral responses are triggered by upregulated carbon fluxes and efflux to biosynthesis, massive induction of protein, ribonucleotide synthesis, membrane proliferation, ER, and RER. Some of the CPE was evident by submitting infected U251 cells to transmission electron microscopy (TEM) analysis. Among them, the compartmentalization and vacuolization of cells infected with DENV3 MG-20 are compatible with the apoptosis mechanism (Fig. 6C). More intense hypertrophy of the endoplasmic reticulum is also observed in the DENV3 MG-20 infection, possibly justified by the high induction of membrane structures supporting viral replication [33]. As can be seen in Fig. 6F and I, there is an intense flow of vesicles filled with cellular and viral molecules such as E protein (as seen in IFI: complex 4G2 antibody -E protein), suggesting that this protein is related to cell-to-cell generating syncytium. Serafino et al. (2003) [34], in their studies, infected human lymphoblastoid (TO.FE) cells culture with hepatitis C virus (HCV) and analyzed CPE using TEM. They also observed Golgi complex and endoplasmic reticulum hyperplasia and viral particles in cells’ cytoplasmic vesicles. Therefore, as DENV and HCV belong to the same family, this phenomenon seems similar to the Flaviviridae family.

The large amount of viral proteins within vesicles of DENV3-MG20-infected U251 cells is a fact that draws attention. Studies have shown that the expression of viral proteins and the regulation of transcription are related to infection-differentially induced ribosomal host proteins [35, 36]. It has been described that flavivirus infections induce a specific expression of the RPLP1/2 ribosomal complex, promoting the accumulation of viral proteins in the early stages of infection [35]. It is also suggested that RPLP1/2 could interact with viral transmembrane domains (including in E protein), and bind to hydrophobic or charged regions, providing stability to the newly formed protein and increasing DENV protein expression [36]. Naturally mutations in charge and hydrophobic protein locations can affect the stability of the three-dimensional structure and result in protein rearrangements, compromising protein folding and stability.

A massive induction of protein expression could also be associated with misfolded proteins and the unfolded protein response (UPR), which serves as an activation mechanism for apoptosis and increased pathogenicity in DENV infection of the liver [37]. We suggest that the cell type, amount of protein expression, and the intense CPE of DENV3 MG-20 observed in U251 cells are responsible for the differences in suggestive apoptotic behaviors. Observing electron-dense particles with the size and appearance of DENV particles confirms that both viruses could infect cells. However, the DENV3 MG-20 isolate demonstrates high protein expression levels in these cells. Figure 6 L depicts a potential site of viral synthesis in the cell, characterized by an abundance of proteins, an enlarged endoplasmic reticulum, and vesicles containing viral particles.

In Diamond et al., 2020 [10], different genotypes of DENV 2 generated divergent effects in cells. Here, we show natural isolates of DENV3 from the same lineage and high similarity, generating distinct profiles of CPE and syncytium in nerve cells. This GIL1 lineage is epidemiologically linked to involvement in the CNS in humans. These distinct pathogenic profiles were also visible in vivo models, in which mice infected with neurovirulent DENV3 MG-20 died, and those infected with DENV3 PV_BR did not show clinical signs of illness [12]. Syncytium formation is a virulence factor, increasing pathogenesis and disease severity. Cell-to-cell fusion allows a fast RNA and viral spread to neighboring cells, possible evasion from the humoral immune response, long-term infection persistence, and increased cytotoxic responses (Fig. 7). These factors could provide an escape from neutralizing antibodies and resistance to possible antivirals and immunobiological agents [33]. Due to location and impacts on chemical-physical properties, we hypothesize that the 62 and 123 amino acids in the DENV3 MG-20 isolate are related to an increased membrane fusion ability in viral infection, making it capable of infecting CNS cells. Additionally, syncytial formation after infection with DENV3 MG-20 could enable the virus to evade the antibody response, facilitating the infection of CNS cells.

## Conclusions

Our study contributes to the understanding the neurological dengue disease. It presents a comparative model of highly similar isolates with the potential to generate more subsidies for a deeper understanding of DENV pathogenesis.

## Data Availability

All data and materials are available under request.
